# Altered pituitary morphology as a sign of benign hereditary chorea caused by TITF1/NKX2.1 mutations

**DOI:** 10.1007/s10048-021-00680-3

**Published:** 2022-01-25

**Authors:** Steffi Thust, Liana Veneziano, Michael H. Parkinson, Kailash P. Bhatia, Elide Mantuano, Cristina Gonzalez-Robles, Indran Davagnanam, Paola Giunti

**Affiliations:** 1 National Hospital for Neurology and Neurosurgery, Queen Square, London, WC1N 3BG UK; 2 Institute of Translational Pharmacology, National Research Council of Italy, Via Fosso del Cavaliere 100, 00133 Rome, Italy; 3 Ataxia Centre, Department of Clinical and Motor Neuroscience, UCL Queen Square Institute of Neurology, London, WC1N 3BG UK; 4 Department of Clinical and Motor Neuroscience, UCL Institute of Neurology, QueenSquare, London, WC1N 3BG UK; 5 Brain Repair and Rehabilitation Unit, UCL Institute of Neurology, QueenSquare, London, WC1N 3BG UK

**Keywords:** Benign hereditary chorea, Brain-lung-thyroid syndrome, Pituitary gland, Pituitary cyst, NKX2.1

## Abstract

Benign hereditary chorea (BHC) is a rare genetically heterogeneous movement disorder, in which conventional neuroimaging has been reported as normal in most cases. Cystic pituitary abnormalities and features of empty sella have been described in only 7 patients with BHC to date. We present 4 patients from 2 families with a BHC phenotype, 3 of whom underwent targeted pituitary MR imaging and genetic testing. All four patients in the two families displayed a classic BHC phenotype. The targeted pituitary MR imaging demonstrated abnormal pituitary sella morphology. Genetic testing was performed in three patients, and showed mutations causing BHC in three of the patients, as well as identifying a novel nonsense mutation of the TITF1/NKX2-1 gene in one of the patients. The presence of the abnormal pituitary sella in two affected members of the same family supports the hypothesis that this sign is a distinct feature of the BHC phenotype spectrum due to mutations in the TITF1 gene. Interestingly, these abnormalities seem to develop in adult life and are progressive. They occur in at least 26% of patients affected with Brain-lung-thyroid syndrome. As a part of the management of these patients we recommend to perform follow-up MRI brain with dedicated pituitary imaging also in adult life as the abnormality can occur years after the onset of chorea.

**Supplementary Information:**

The online version contains supplementary material available at 10.1007/s10048-021-00680-3.

## Introduction

Benign hereditary chorea (OMIM: #118700) is a rare possibly dominantly inherited hyperkinetic movement disorder, with underlying heterogeneous genetic causes. The first gene that has been identified is NKX2-1, also known as TITF-1, on chromosome 14q13.3 coding for the thyroid transcription factor 1 [1]. Mutations in a second gene, ADCY5, coding for the adenylate cyclase 5, have been found to be another cause of benign hereditary chorea [2]. Recently, mutations in PDE10A, encoding an enzyme involved in the hydrolysis/degradation of cAMP and cyclic guanosine monophosphate (cGMP), have been reported in patients with infantile/childhood‐onset chorea [3]. *TITF1/NKX2-1* encodes for a thyroid-specific enhancer-binding protein (*T/EBP*), which plays a regulatory role in thyroid, brain, and lung organogenesis. Hence, the underlying genetic defect may manifest as “Brain-Lung-Thyroid syndrome” (BLT, OMIM: #610978), characterized by a broad phenotypical spectrum, including neurological abnormalities, congenital hypothyroidism, infant respiratory distress syndrome, recurrent pulmonary infections, or interstitial lung disease [4–6]. Several TITF1/NKX2-1 mutations have been identified, in which brain MR imaging was unremarkable. However, the presence of pituitary abnormalities has been described in a small proportion of BHC patients, 7 to date [7–10] This subset of patients raised the question of whether these findings were incidental or represented part of the variable BHC phenotype.

### TITF1 in pituitary development

The pituitary gland originates from two embryonic tissues: the oral ectoderm for the adenohypophysis (the anterior and intermediate lobes) and the neural ectoderm for the neurohypophysis (the posterior lobe).

The anterior, intermediate, and posterior lobes of the pituitary gland function as three separate endocrine organs, each characterized by distinct cell populations, secretory products, and regulatory mechanisms.

The anterior lobe is a highly specialized tissue that contains a functionally diverse population of cell types committed to synthesize and secrete five different hormones during development [1]. The intermediate lobe is rudimentary in humans but produces MSH. Pituitary cysts are generally located in this portion. The posterior lobe releases oxytocin and vasopressin from axon terminals that originate in cell bodies located in the hypothalamus [11]. Pituitary development occurs in successive steps that are controlled by several transcription factors having a distinct temporal and spatial expression pattern. They interact with each other and with additional exogenous and endogenous signals to control cell determination and differentiation [11]. TITF1 is one of the numerous transcription factors involved in the development of the pituitary and acts at a very early stage, during the formation of the posterior lobe. In spite of TITF1 not being expressed in the intermediate and anterior lobe, in the TITF1 null mouse, the pituitary is completely missing, suggesting that the presence of the posterior lobe and/or TITF1 gene expression is required for full development of the anterior and intermediate pituitary [11–13]. Interestingly, TITF1 plays a pleiotropic function having various roles in different stages of the development and differentiation of several organs, such as lung, brain, thyroid, and pituitary. The pleiotropic functions are due to the action of two different activation domains and to specific post-translational modifications. [12]

Here, we present pituitary imaging in three patients, in whom recently a novel TITF1 mutation was discovered and all of which had an altered sella morphology. [8]

This work was approved by REC 04/Q0505/21.

### Clinical features

The neurological presentation of BHC is typically in childhood before the age of 5 years, although age of onset may be variable from infancy to adolescence [14, 15]. Typical signs include early hypotonia and delayed motor development, followed by walking difficulties, ataxia, with frequent falls, and usually later onset of chorea [16, 17]. Typically, cognition and speech are preserved, although cases of cognitive impairment and even psychiatric disturbance have been reported [18–21]. A progressive course in BHC is rare, although this has been described, and life expectancy lies within the normal range [22, 23]. Despite the original name “benign hereditary chorea,” only 13% presented with isolated chorea [24]. Some patients exhibit dystonia, myoclonus, tremor, ataxia, and dysarthria, which can make the clinical distinction between BHC and other neurological syndromes challenging [25–27]. The 50% of patients with NKX2-1 mutations presented with a combination of neurological, pulmonary, and thyroid symptomatology. Thus, the “benign” phenotype initially described is actually uncommon [28]. In light of the varied manifestations of heterozygous mutations in NKX2-1, some authors suggest that the term hereditary benign chorea should be replaced by *NKX2-1-related disorders*. [29]

## Materials and methods

### Family 1

This 49-year-old subject presented in infancy with delayed milestones before being diagnosed with cerebellar ataxia at the age of 2. The patient had lifelong balance problems with onset of jerky movements in adolescence. Over the last 10 years, some worsening in choreiform movements was noticed, as well as an increased frequency of falls. Physical examination revealed mild gait ataxia with dystonic posturing of the hands, choreic jerks, and dystonic movements of the head and shoulders. Mild ocular apraxia was noted as well as a degree of dysdiadochokinesia due to intrusion of involuntary movements.

We also examined a 26-year-old patient who is the offspring of the above described subject (case 1). Similar to case 1, delayed motor development was present, with independent walking achieved at the age of 2. Balance problems and frequent falls were present throughout childhood despite receiving intensive physiotherapy, with mild spontaneous improvement in adulthood. Examination revealed choreiform movements of the head and legs with no other neurological abnormality.

There are no other affected family members.

### Family 2

The third patient arrived to our clinics at the age of 35. The medical history showed delayed motor milestones. At birth, twitching movements of the limbs spreading throughout the body were noticed. Ataxia and falls were common in childhood and slowly improved. On examination, gait was impaired by both chorea and dystonia. One of their children was also born prematurely, with difficulties in feeding and delayed motor milestones. The affected child continued to experience falls and problems with walking, and had similar examination findings to the affected parent.

The pedigree of both families is detailed in Supplementary Figure 1.

### Molecular genetics

Screening for mutations in the NKX2.1 gene, genomic DNA was amplified by PCR using primer pairs as described by Breedveld et al [1]. The obtained DNA fragments, all the three NKX2.1 coding exons, were sequenced with the Sanger method by Eurofins Genomics service (https://eurofinsgenomics.eu/en/eurofins-genomics-genomic-services-by-experts/).

Screening for mutations in the NKX2.1 gene was performed in all affected subjects since mutations of this gene are the main cause of BHC phenotype. No other gene was screened because the diagnostic workflow suggests to screen the NKX2.1 gene as the first step. Mutations of other genes, as ADCY5 and PDE10A, are rarer.

## Results

### Family 1

#### Laboratory and genetic results

The 49-year-old patient had hypothyroidism, but otherwise had normal pituitary function tests. A biochemical profile performed on the 26-year-old patient showed a mildly reduced prolactin level and marginally raised thyroid-stimulating hormone (normal free T4 level).

Both parent and child showed the heterozygous nucleotide substitution NM_001079668.2:c.631A>T, which results in the change of a lysine residue for a stop codon at position 211, NM_001079668.2(NKX2-1_i001):p.(Lys211*) (nomenclature according to HGVS format). This de novo mutation was previously reported by our group [8].

### Imaging results

**Figure 1:**
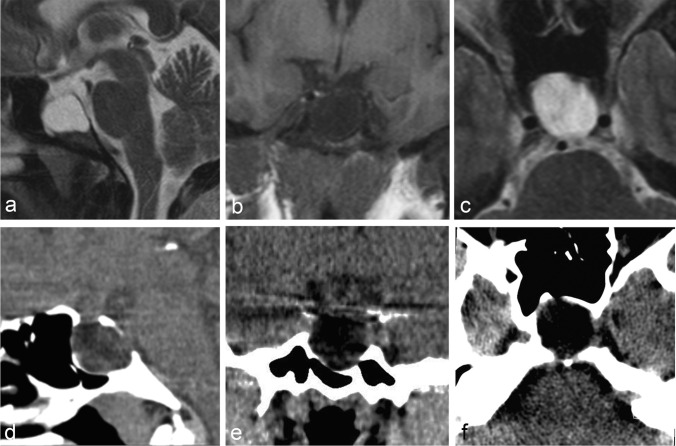
Non-contrast MRI (panels **a**–**c**) of the pituitary gland with sagittal (**a**) and axial (**c**) T2-weighted as well as coronal T1-weighted dedicated 3-mm thin-sections from case 1, demonstrating thin rim of pituitary tissue along the anterior and inferior aspects of the enlarged CSF-filled sella turcica. Non-contrast multiplanar reformatted CT images (panels **d**–**f**) in the corresponding sagittal (**d**), coronal (**e**), and axial (**f**) planes demonstrating smooth remodelling of the osseous boundaries of the sella turcica with no evidence of bony erosion.

Targeted high-resolution pituitary MR imaging of the 49-year-old subject demonstrated marked expansion of the CSF-filled sella turcica (Figure 1) with a slender rim of pituitary gland tissue draped along the anterior wall and floor of the sella. Corresponding CT imaging revealed hinning of the pituitary fossa bone margins without evidence of bone destruction. The remaining midline brain structures were normal on imaging.

**Figure 2:**
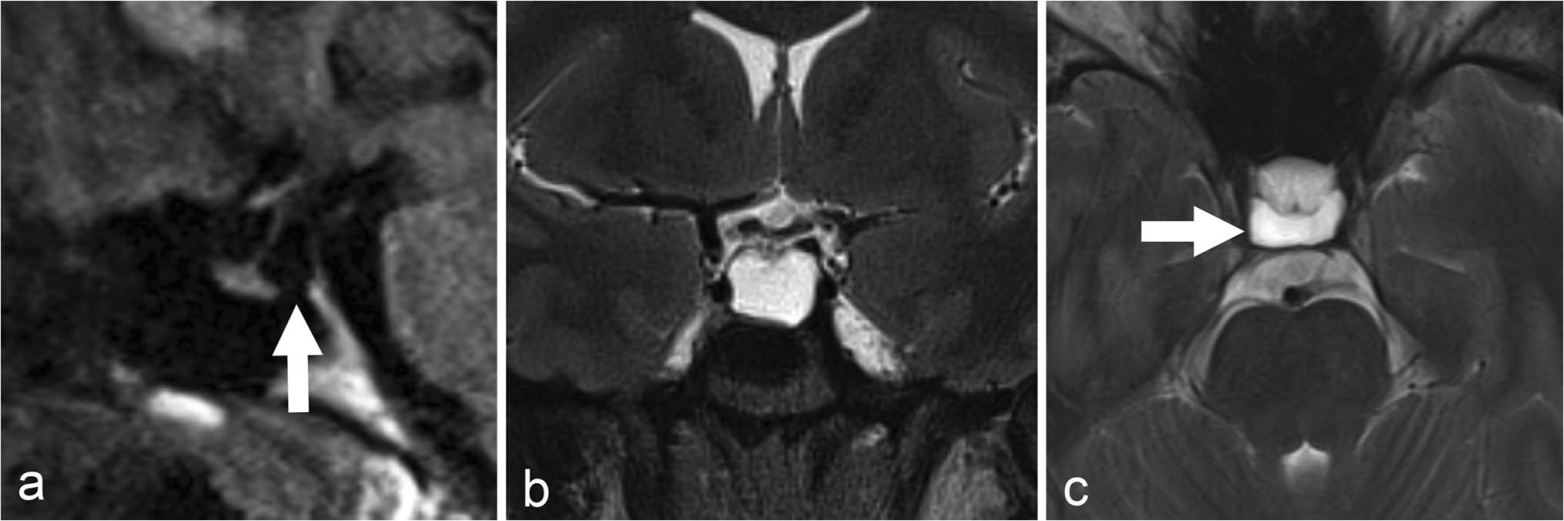
Non-contrast MRI (panels **a**–**c**) of the pituitary gland with sagittal (**a**) T1-weighted as well as coronal (**b**) and axial (**c**) T2-weighted dedicated 3-mm thin-sections from case 2. The imaging demonstrates predominantly dorsal expansion of the intrasellar space (white arrow in panel **a**) with anterior deviation of the infundibulum and relative flattening of the superior contour of the pituitary gland.

Whole brain MR imaging supplemented by dedicated thin-section (3 mm) pituitary views of the 26-year-old patient showed marked cystic expansion of the sella turcica with mild anterior displacement of the pituitary stalk and gland (Figure 2).

### Family 2

#### Laboratory and genetic results

Both parent and child carry a heterozygous mutation NM_001079668.2:c.605A>G which is predicted to change a glutamine to an arginine NM_001079668.2(NKX2-1_i001):p.(Gln202Arg). This is an evolutionarily highly conserved residue, not reported in databases of polymorphic variants.

In silico analysis of the missense mutation was performed using VARSOME software (https://varsome.com/) which is an annotation tool and search engine for human genomic variants, and a platform enabling the sharing of knowledge on specific variants According to all the 30 databases involved in VARSOME evaluation, this mutation results likely pathogenic (https://varsome.com/variant/hg19/NKX2.1%3AQ202R?annotation-mode=germline).

Furthermore, a functional characterization of a different mutation of the same amino acid (NM_001079668.2:c.606G>C; p.Gln202His) showed that it causes a decrease in the DNA-binding activity, leading to a loss of protein function [30].

### Imaging results

**Figure 3:**
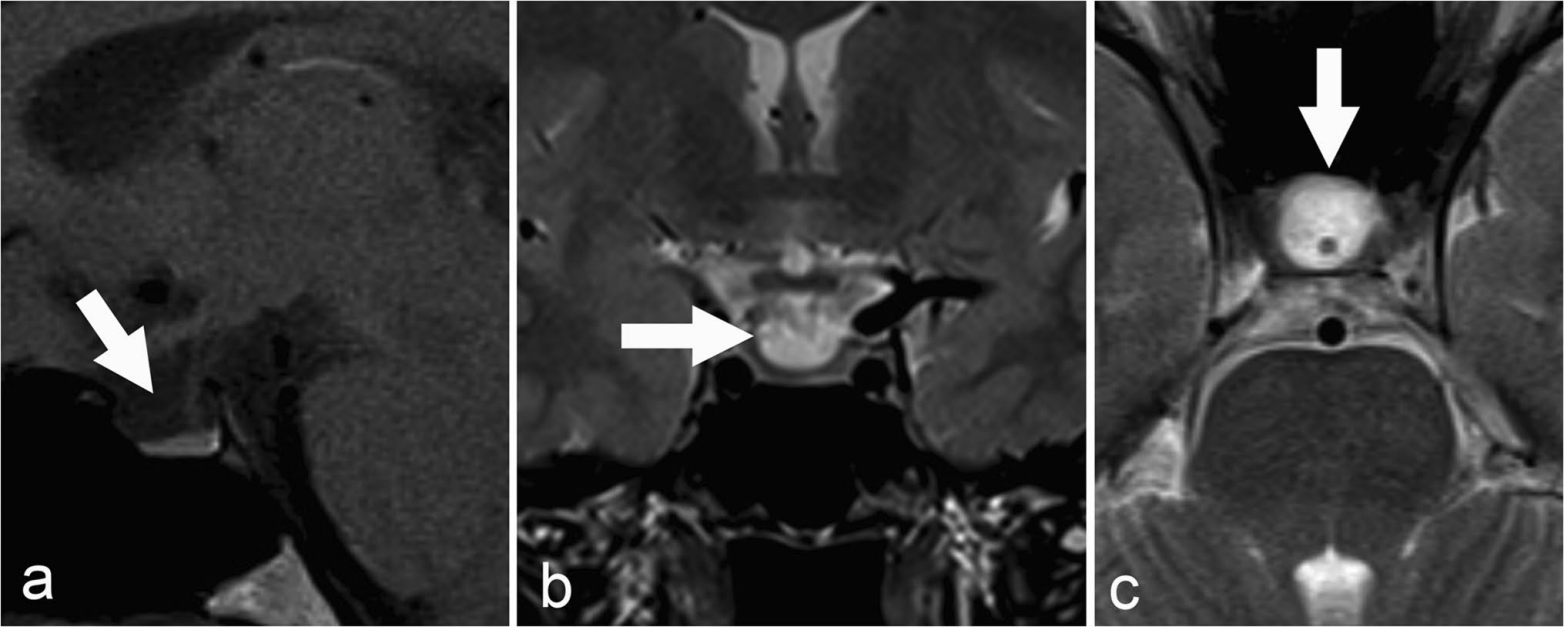
Non-contrast MRI (panels **a**–**c**) of the pituitary gland with sagittal (**a**) T1-weighted as well as coronal (**b**) and axial (**c**) T2-weighted dedicated 3-mm thin-sections from case 3. There is subtle expansion of the anterior recess of the sella to thicker with flattening of the superior contour of the adenohypophysis (white arrows in panels **a**–**c**).

The parent’s MR imaging demonstrated subtly altered morphology of the intrasellar structures with cystic enlargement of the CSF space anterior to the pituitary gland and flattening of the adenohypophyseal surface (Figure 3). No other intracranial abnormality was present.

#### Discussion

The relationship of TITF-1 deficiency with abnormal basal ganglia development, in particular impaired striatal differentiation, has been well established in mice [1, 11]. Loss of striatal interneurons was demonstrated in a human pathological specimen of a patient with BHC [31]. TITF-1 seems to play a critical role for the interneuron specification of medial ganglionic eminence cells [32], and the regulation of the direction of the migrating interneurons [33]. Importantly, TITF-1 has also been shown to promote development of the posterior pituitary and hypothalamus [34, 35]. In fact, in a study by Kimura et al., homoygous *TITF1* knockout mice were born dead and lacking lungs, thyroid, and pituitary gland. [11]

Krude et al. described two patients with a posterior pituitary cystic mass [7]. Accornero et al. presented a single case of a patient with pituitary stalk duplication and changes in the basal ganglia, caused by a deletion on chromosome 14 harboring *TITF1*. [35] Salvatore et al. identified features of “empty sella” in two adult patients, whereby the abnormality was more marked in the parent who had longer disease duration [5]. Balicza et al. reported a family where two patients with stop mutation of NKX2-1 gene had “empty sella” on MRI and pituitary hormone deficiencies [10]. The imaging features in the latter publication are strikingly similar to the patients in our series, supporting the hypothesis of progression over time. [8]

The three English cases presented here represent mutations in exon 3 of the *TITF1* gene encoding for the homeodomain of TTF-1, where most point mutations associated with BHC are located. All three patients demonstrate altered sella turcica morphology ranging from subtle flattening of the superior gland surface (case 2 and 3) to a large posterior intrasellar cyst (case 1). The mechanism for the development of these changes is unclear, but could represent a combination of congenital maldevelopment and acquired pathology, possibly as a result of local CSF pressure, given the presence of bony thinning in the oldest patient, who is most severely affected. Interestingly, one group described intrasellar cyst formation unrelated to BHC in the context of a persistent embryonal infundibular recess proven at surgery [36]. The exact mechanism for the development of sella abnormalities in BHC remains still unknown. Many mutations have been identified in TITF1 gene (large gene deletions and missense and nonsense mutations spanning the entire gene), but there is no relationship between the type of mutation and the severity of the phenotype. Clinical heterogeneity and incomplete penetrance of the disease cannot be predicted only on the basis of the mutation type. Environmental factors, tissue factors, and genetic background could influence the clinical phenotype of BHC patients [1]. Severity and organ involvement may also vary in a single pedigree [37]. Chorea can be the predominant or the only symptom associated with TITF1 gene mutations [25, 38]. With such clinical heterogeneity of the disease, we cannot exclude cases in which pituitary cysts or pituitary malformations could be the only symptom associated to TITF1 gene mutations. Therefore, it could be relevant looking for mutations in TITF1 gene in patients in which pituitary cysts or pituitary malformations have been diagnosed.

Among 98 cases of TITF1/NKX2-1 mutations published to date (see Table 1), most report normal imaging findings or do not feature imaging descriptions. A few groups showed subtle reduced basal ganglia tracer uptake in BHC on nuclear medicine imaging, but concluded that conventional neuroimaging is typically normal [19, 39]. Pituitary-sella abnormalities have only been reported in 7 families (7.1%). A brain MRI scan is reported for 77 patients with NKX2-1 mutations. Furthermore, among these patients, the frequency of pituitary abnormalities reaches 13%, and 26% in the cases of NKX2-1-related disorders, suggesting that pituitary malformations are present as sign of the disease.

**Table 1.** Mutations in TITF1/NKX2.1 with neurological, thyroid, lung, or pituitary involvement

**Mutation**	**Transmission**	**Brain**	**Thyroid**	**Lung**	**Brain MRI**	**N. patients with brain MRI**	**N. patients with pituitary abnormalities**	**Notes**	**References**
§p.M59AfsX40	De novo	+	–	+	NR				McMichael, 2013 [40]
§p.Y98X	De novo	+	+	–	NR				Tübing, 2018 [41]
p.Y98X	AD	+	+	+	–	2			Nakamura, 2012 [42]
p.Q107X	AD	+	–	–	–	4			Sempere, 2013 [43]
p.G115AfsX10	De novo	+	–	+	–	1			Parnes, 2018 [28]
p.Y116fsX323	De novo	+	+	+	–	1			Pohlenz, 2002 [44]
p.Y116X	AD	+	–	–	NR				Gras, 2012 [45]
p.C117X	?	+	+	+	NR				Krude, 2002 [7]
p.P129fsX307	De novo	+	–	+	NR				Hamvas, 2013 [46]
p.Y130X	De novo	+	+	+	–	1			Parnes, 2018 [28]
p.Y130X	De novo	+	+	+	–	1			Iodice, 2019 [47]
p.T133NfsX306	De novo	–	+	+	–	1			Parnes, 2018 [28]
§p.W143X	De novo	+	+	–	+	2	2	Empty sella	Balicza, 2018 [10]
p.Y144X	?	+	+	–	NR				Teissier, 2012 [48]
p.Y144X	?	+	+	+	NR				Hamvas, 2013 [46]
§p.R157AfsX7	De novo	+	+	–	–	1			Milone, 2019 []
c.463 + 1_463 + 4del	AD	+	+	–	NR				Gras, 2012 [45]
c.463 + 1G > A	De novo	+	+	+	–	1			Fons, 2012 [49]
c.464-9C > A	AD	+	+	–	–	6			Konishi, 2013 [50]
c.464-1G > A	De novo	+	+	+	–	1			Barreiro, 2011 [51]
c.464-2A > C	AD	+	–	–	–	2			Asmus, 2007 [25]
c.464-2A > T	AD	+	–	NR	–	1			Kleiner-Fisman,2003 [31]
c.464-2A > G	AD	+	+	+	–	2			Doyle, 2004 [52]
c.464-2A > G	De novo	+	+	+	–	1			Carrè, 2009 [24]
p.S163fsX2	De novo	+	+	+	NR				Gras, 2012 [45]
p.S175X	AD	+	+	+	+	2	2	Empty sella	Ferrara, 2008 [53]; Salvatore, 2010 [5]
p.P185fsX250	De novo	+	+	+	NR				Hamvas, 2013 [46]
p.P187fsX196	De novo	+	+	+	–	1			Nagasaki, 2008 [54]
p.R195fsX32	AD	+	+	+	_	1			Nettore, 2013 [55]
p.R195W	De novo	+	+	+	NR				Hamvas, 2013 [46]
p.L197P	?	+	–	+	NR				Hamvas, 2013 [46]
p.F198L	AD	–	–	+	NR				Hamvas, 2013 [46]
p.F198L	?	–	–	+	NR				Hamvas, 2013 [46]
p.F198L	?	–	–	+	NR				Hamvas, 2013 [46]
p.S199X	?	+	+	–	NR				Krude, 2002 [7]
§p.Q202H	De novo	+	+	–	–				Provenzano, 2016 [30]
p.Q202R	De novo	+	–	–	+	1	1	Empty sella	*Present paper*
p.E205X	AD	_	–	–	–	3			Asmus, 2005 [56]
p.L206V	De novo	+	–	–	–	1			Gras, 2012 [45]; Carrè, 2009 [24]
p.R208X	AD	+	–	–	–	1			Provenzano, 2008 [57]
p.R209P	De novo	+	+	–	_	2			Williamson, 2014 [58]
p.K211X	De novo	+	+	–	+	2	2	Cystic mass, empty sella	Veneziano, 2014 [8]
p.Y215D	De novo	+	+	–	NR				Gras, 2012 [45]
p.S217X	AD	+	+	–	NR				Glik, 2008 [20]
p.L224R	AD	+	+	–	NR				Gras, 2012 [45]
p.L224R	De novo	+	+	+	NR				Koht, 2016 [59]
p.A225fsX228	De novo	+	–	–	NR				Krude, 2002 [7]
p.L230P	De novo	+	+	–	–	1			Iodice, 2019 [47]
p.P233L	De novo	+	+	–	NR				Carrè, 2009 [24]
p.V235P	De novo	+	+	+	+	1	1	Cystic mass	Krude, 2002 [7]
p.V235P	AD	+	+	–	–	2			Uematsu, 2012 [18]
p.I237F	De novo	NR	+	+	NR				Maquet, 2009 [60]
p.I237M	De novo	–	+	+	NR				Gillet, 2013 [61]
p.W238L	AD	+	NR	NR	–	1			Breedveld, 2002 [1]
p.W238CfsX9	De novo	+	+	–	–	1			Iodice, 2019 [47]
§p.W238S	De novo	+	+	–	–				Provenzano, 2016 [30]
p.Q240P	De novo	+	+	–	–	1			Gras, 2012 [45]; Carrè, 2009 [24]
p.R243S	AD	+	NR	NR	–	1			Breedveld, 2002 [1]
p.R243P	AD	+	–	–	NR				Gras, 2012 [45]
p.Y244X	AD	+	+	–	NR				Gras, 2012 [45]
p.Q249X	AD	+	–	–	–	2			Costa, 2005 [62]
p.D252VfsX187	De novo	+	+	+	–	1			Parnes, 2018 [28]
p.G266del	AD	+	NR	+	–	1			Zorzi, 2008 [63]
p.G269_271dupGGGa	?	+	–	+	NR				Hamvas, 2013 [46]
p.274_280del7aa and p.G273fsX152	?	+	+	+	NR				Hamvas, 2013 [46]
p.A280fsX161	AD	+	+	+	NR				Teissier, 2012 [48]
p.P291R	De novo	+	+	–	–	1			Iodice, 2019 [47]
p.L293del	De novo	+	+	+	NR				Gras, 2012 [45]
p.G303fsX77	AD	+	NR	NR	–	1			Breedveld, 2002 [1]
p.A306fsX350	AD	+	+	–	–	2			Moya, 2006 [37]
p.Q327fsX121	De novo	+	+	+	–	1			Willemsen, 2005 [4]
p.A327GfsX52	De novo	+	+	+	NR				Shetty, 2014 [64]
p.A329GfsX108	De novo	+	+	+	NR				Hermanns, 2018 [65]
p.A333RfsX132	De novo	+	+	+	–	1			Tozawa, 2016 [66]
p.H349fsX90	De novo	+	+	+	NR				Hamvas, 2013 [46]
p.Q357fsX24	AD	+	–	–	–	3			Mahajnah, 2007 [39]
p.S366fsX67	?	–	+	+	NR				Hamvas, 2013 [46]
p.T389fsX52	?	+	+	+	NR				Hamvas, 2013 [46]
del 14q13-q21	De novo	NR	+	+	–	1			Devriendt , 1998 [67]
del 14q12-q13.3	De novo	+	+	+	–b	2			Iwatani, 2000 [68]
del 14 1.2 MB	De novo	+	NR	NR	–	1			Breedveld, 2002 [1]
del 14 1.2 MB	AD	+	+	NR	+	2	1	Stalk duplication	Accornero, 2010 [35]
del 14q11.2-q13.3	?	+	+	+	+	1	1	Cystic mass	Krude, 2002 [7]
del 14q13	De novo	+	+	+	–	1			Carrè, 2009 [24]
del 14 0.9 MB	AD	+	+	+	–	3			Devos, 2006 [69]
del 14q12-q13	De novo	+	+	+	–	1			Uematsu, 2012 [18]
del 14q13.2-q22.1	De novo	+	–	–	NR				Gras, 2012 [45]
del 14q13.2-q21.2	De novo	+	+	–	NR				Gras, 2012 [45]
del 14q13.3	De novo	+	+	–	NR				Gras, 2012 [45]
del 14q13.1-q21.1	De novo	+	+	+	NR				Hamvas, 2013 [46]
del 14q13.3	?	+	+	+	NR				Hamvas, 2013 [46]
del 14q13.3-q21.1	De novo	+	+	+	NR				Hamvas, 2013 [46]
del 14q13.1-q21.1	De novo	+	+	+	NR				Hamvas, 2013 [46]
DEL ex1-2	?	+	+	+	NR				Hamvas, 2013 [46]
del 14q13.2-q21.1	De novo	+	+	–	–	1			Dale, 2012 [70]
del 14q13.3	AD	+	+	+	NR				Teissier, 2012 [48]
del 14q13.3	De novo	+	+	–	NR				Teissier, 2012 [48]
del 14q13.2-q21.1	De novo	+	+	+	–	1			Villafuerte, 2018 [71]

*AD* autosomal dominant, *NR* not recorded, *?* unknown, *§* these mutations, published according to the short NKX2.1 isoform, have been reported to the long isoform ref seq NM_001079668.3, NP_001073136.1.

The families reported in the literature showed that the pituitary abnormality is worse in patients with a longer disease duration [8, 9]. Altered pituitary-sella morphology could be an under-recognized phenomenon related to loss of function of NKX2-1 gene. To date, no pituitary abnormalities are reported in the literature in carriers of ADCY5 and PDE10A gene mutations. ADCY5 mutations are known to be more related to atrophy in the frontoparietal cortex and thalamus [72], while PDE10A mutations are more associated with increased signal intensity and atrophy within the striatum (Table 2). [3]

**Table 2.** MRI characteristics associated with TITF-1, ADCY5, and PDE10A mutations.

**Gene**	**Gene product**	**Inheritance**	**Age of onset**	**MRI characteristic features**
TITF-1/NKX2-1	Thyroid transcription factor 1	AD/de novo	Childhood/adulthood	Altered sella turcica morphology
ADCY5	Adenylate cyclase 5	AD/de novo	Infancy to childhood	Frontoparietal cortex and thalamus atrophy
PDE10A	Phosphodiesterase 10A	De novo/AD/AR	Infancy to childhood	Bilateral striatal hyperintensities and bilateral striatal atrophy

In conclusion, dedicated pituitary imaging should therefore be considered in patients presenting with a clinical phenotype of BHC to guide the diagnosis. In addition, all patients with no brain MRI abnormalities during the first investigations should undergo regular follow-up at a couple of years’ intervals. Patients with known abnormalities in the pituitary sella should undergo a routine ophthalmological evaluation including visual fields. Moreover, to rule out pituitary dysfunction, a complete pituitary hormones assay should be routinely performed. We showed the usefulness of the brain MRI with dedicated imaging to the pituitary gland in BHC patients and the value of follow-up imaging in those patients with no changes on the investigation at baseline. The presence of these abnormalities could predict the genetic diagnosis of TTF1-related BHC. Our findings could be useful to improve genetic and neurological counselling of BHC and should be embedded in clinical guidelines.

## Supplementary Information

ESM 1 Pedigrees of the affected individuals. S1a. Family 1: The affected index case (I.1, arrow) had delayed milestones, cerebellar ataxia jerking, choreiform movements and dystonia. Their child (II.1) suffered from delayed milestones and displayed choreiform movements on examination. S1b. Family 2: The affected index case (I.1, arrow) had delayed motor milestones, chorea, dystonia and gait difficulties. Their child (II.1) was prematurely born, and also had a delay in their motor milestones, as well as chorea, dystonia and gait problems.(PDF 39 kb)

## Abbreviations

ADCY5: Adenylate cyclase 5
BHC: Benign hereditary chorea
BLT: Brain-lung-thyroid (syndrome)
cAMP: Cyclic adenosine monophosphate
cGMP: Cyclic guanosine monophosphate
CSF: Cerebrospinal fluid
CT: Computerized tomography
HGVS: Human Genome Variation Society
MRI: Magnetic resonance imaging
MSH: Melanocyte-stimulating hormone
NKX2-1: NK2 Homeobox 1
PDE10A: Phosphodiesterase 10A
T/EBP: Thyroid-specific enhancer-binding protein
TITF-1: Thyroid transcription factor 1
