# Supplementary figures and images for: Altered pituitary morphology as a sign of benign hereditary chorea caused by TITF1/NKX2.1 mutations
Source: Neurogenetics. 2022 Jan 25;23(2):91–102. doi: 10.1007/s10048-021-00680-3 (PMC8960566; doi:10.1007/s10048-021-00680-3)

**Supplementary Figure 1.**

a.

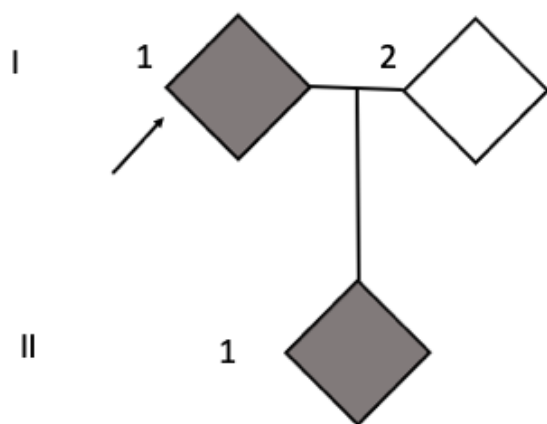

b.

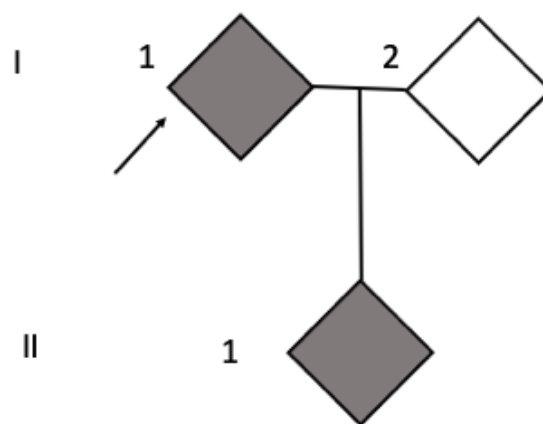

Supplement: Supplementary file 1 — Pedigrees of the affected individuals. S1a. Family 1: The affected index case (I.1, arrow) had delayed milestones, cerebellar ataxia jerking, choreiform movements and dystonia. Their child (II.1) suffered from delayed milestones and displayed choreiform movements on examination. S1b. Family 2: The affected index case (I.1, arrow) had delayed motor milestones, chorea, dystonia and gait difficulties. Their child (II.1) was prematurely born, and also had a delay in their motor milestones, as well as chorea, dystonia and gait problems.(PDF 39 kb) [file 10048_2021_680_MOESM1_ESM.pdf]
